# Could HIF-1α be a novel biomarker for the clinical course and treatment of pulmonary embolism?

**DOI:** 10.3906/sag-1908-93

**Published:** 2020-06-23

**Authors:** Buğra KERGET, Dursun Erol AFŞİN, Alperen AKSAKAL, Seda AŞKIN, Ömer ARAZ

**Affiliations:** 1 Department of Pulmonary Diseases, Health Sciences University Erzurum Regional Education and Research Hospital, Erzurum Turkey; 2 Department of Pulmonary Diseases, Ağrı State Hospital, Ağrı Turkey; 3 Department of Pulmonary Diseases, School of Medicine, Atatürk University, Erzurum Turkey; 4 Department of Biochemistry, School of Medicine, Atatürk University, Erzurum Turkey

**Keywords:** HIF-1 alpha, pulmonary thromboembolism, hypoxia

## Abstract

**Background/aim:**

Pulmonary embolism (PE) is associated with high morbidity and mortality rates if not diagnosed and treated rapidly. The aim of our study was to investigate the relationship between levels of hypoxia-induced factor-1 alpha (HIF-1α) and clinical course and prognosis in patients with intermediate low-risk, intermediate high-risk, and high-risk PE.

**Materials and methods:**

The study included 240 subjects in 4 groups: a healthy control group (n = 60, mean age = 60 ± 15.2, female/male = 30/30 ), intermediate low-risk PE group (n = 60, mean age = 60 ± 12,5, female/male = 27/33), intermediate high-risk PE group (n = 60, mean age = 61,4 ± 14,8, female/male = 36/24), and high-risk PE group (n = 60, mean age = 62,3 ± 15, female/male = 33/27). Plasma HIF-1α levels were measured using commercial enzyme-linked immunosorbent assay (ELISA) kit.

**Results:**

Comparison of HIF-1α levels revealed a statistically significant difference between the groups in proportion to clinical scoring (P = 0.001 for all). Comparison of initial HIF-1α and troponin levels in intermediate high-risk PE patients given thrombolytic therapy and those treated with enoxaparin sodium showed that HIF-1α levels were significantly higher in the group that received thrombolytic therapy (P = 0.001), while there was no difference in troponin levels (P = 0.146).

**Conclusion:**

HIF-1α can be used in the PE clinical risk stratification and monitoring of PE and may also serve as a valuable early indicator in intermediate high-risk PE, for which early reperfusion therapy is important.

## 1. Introduction

Pulmonary embolism (PE) is usually an early complication of deep venous thrombosis (DVT) and can cause high morbidity and mortality rates if not diagnosed and treated early [1]. The etiology of venous thromboembolism can involve genetic risk factors as well as acquired risk factors such as malignancy, pregnancy, oral contraceptive use, immobility, advanced age, and most importantly, prior surgery [2].

The commonly used biomarkers troponin, brain natriuretic peptide (BNP), and N terminal-proBNP (NT-proBNP) are used to classify cases of intermediate PE as low- or high-risk. Right ventricular dilatation due to PE increases myocardial oxygen demand of the right ventricle. Microinfarcts resulting in malperfusion in the right ventricle cause troponin-I release from the myocardium as an indicator of cell damage, while increased levels of HIF-1α and especially VEGF improve the existing pathology [3]. 

Right ventricle dilation stimulates the myocardial tissue and vascular bed to synthesize various cytokines, especially hypoxia inducible factor-1α (HIF-1α), to regulate oxygenation and promote neovascularization. HIF-1α induces vascular endothelial growth factor (VEGF) synthesis to initiate vascularization, while glutathione peroxidase activity is also increased to prevent reperfusion injury [4]. VEGF, the main inducer of which is HIF-1α, plays an important role in thrombus organization and thrombolysis. In addition to inhibiting platelet aggregation by causing an increase in nitric oxide levels, it also promotes the conversion of plasminogen to plasmin (which is thrombolytic) by increasing serine protease, urokinase, and tissue plasminogen activity [5,6]. In studies using rat models of experimental PE, it was determined that HIF-1α was expressed at a higher level than in the control group and that this synthesis primarily occurred in the myocardial tissue and vascular bed [3,7]. 

Cardiac biomarkers are still not completely reliable indicators of disease course; therefore, the aim of the present study was to determine the relationship between HIF-1α levels and clinical scoring and course in patients with PE.

## 2. Materials and methods

### 2.1. Study population

The study included 180 PE patients who received inpatient treatment in the chest diseases department of our hospital between January and June 2019. Before initiating this prospective observational study, ethics committee approval was obtained from the Erzurum Regional Education Research Hospital (BEAH KAEK 2019/10-104). This study has been performed in accordance with the ethical standards laid down in the 1964 Declaration of Helsinki and its later amendments or comparable ethical standards. 

All patients with clinical suspicion of PE (major risk factors, clinical signs) underwent chest x-ray, electrocardiogram (ECG), and echocardiographic (ECHO) evaluation. Those with a high clinical suspicion of PE underwent CT angiography with a 16-slice multidetector CT to confirm the diagnosis.

Clinical and laboratory evaluations, including physical examination, blood pressure, heart rate, complete blood count, and oxygen saturation, were performed before and after thrombolytic therapy. ECG examination and interpretation were done before thrombolytic therapy only.

High-risk PE (n = 60) was defined as sustained hypotension (systolic arterial pressure < 90 mmHg or a drop in systolic arterial pressure of at least 40 mmHg for at least 15 min) and cardiogenic shock (including an altered level of consciousness, oliguria, or cool, clammy extremities). Patients with right ventricular dilatation and troponin I elevation without hypotension or signs of shock were classified as intermediate high-risk PE (n = 60), while those with right ventricular dilatation but no troponin I elevation were considered intermediate low-risk PE (n = 60). Sixty healthy volunteers who did not meet any of the study exclusion criteria and had no history of PE were selected as the control group.

Patients with high risk PE who were under 65 years of age were given alteplase as a 10 mg bolus followed by 90 mg infusion over 2 h, while patients over 65 years of age received half-dose alteplase. Starting immediately after thrombolytic therapy, enoxaparin sodium was administered at 12-h intervals. Patients with intermediate PE received enoxaparin sodium therapy at 12-h intervals. Eighteen patients in the intermediate high-risk PE patient group developed hypotension and shock in the first 72 h and were administered thrombolytic therapy as per the high risk PE protocol.

### 2.2. Exclusion criteria

Patients with history of myocardial infarction and associated treatment in the past 3 months, severe hypoxic respiratory failure, PE with concomitant intracranial hemorrhage, diagnosed malignancy, and known history of right heart dilation were not included in the study.

### 2.3. Blood and serum samples

Computed tomography (CT) and ECG were performed and troponin-I and HIF-1ɑ were assessed before and at the end of treatment for all patients.

### 2.4. Measurement of biochemical markers

Venous blood samples were obtained from all patients from an antecubital vein after at least 15 min of semisupine rest. All blood samples were collected into tubes containing ethylenediaminetetraacetic acid (EDTA) to prevent coagulation. Troponin I concentrations were measured by chemiluminescent immunoassay using an Immulite 2500 (Siemens Medical Solutions, Erlangen, Germany). HIF-1α was measured by enzyme-linked immunosorbent assay (Elabscience human elisa kit, UK).

### 2.5. Statistical analysis

The study data were analyzed using SPSS v.20.0 software. Categorical variables were expressed as number and percentage; numerical variables were expressed as mean and standard deviation. The Kolmogorov–Smirnov test was used to test the normality of data distributions. Independent-samples t-test was used to compare baseline HIF-1α and troponin-I levels in intermediate high-risk PE patients according to treatment status and in surviving and nonsurviving high-risk PE patients. For all groups, baseline and 72-h HIF-1α and troponin-I levels, systolic PAP levels, and hospital length of stay were compared using the Kruskal–Wallis test, after which significant parameters were compared with ANOVA with post hoc Tukey test. ROC curve analysis was used to calculate the sensitivity and specificity of baseline HIF-1α and troponin-I levels in patients with and without high-risk PE. Correlations between baseline HIF-1α and troponin-I levels and systolic PAP were evaluated using the Pearson correlation analysis. Results with a P-value of <0.05 were considered statistically significant. 

## 3. Results

The mean age of the patient groups was 63 ± 14.3 years. There was no statistically significant difference in mean age between the study groups. The mean age of the control group was 60 ± 15.2 years. There was no significant age difference between patients and controls.

In the high-risk PE group, 9 of the 60 patients died within the first 72 h and 12 patients died after 72 h. Nine patients in the intermediate high-risk PE group died after 72 h. Causes of death in the high-risk PE group were intracerebral or intraabdominal hemorrhage for 3 patients and hypoxic respiratory failure for 18 patients. In the intermediate high-risk PE group, 3 patients died due to intracerebral hemorrhage following thrombolytic therapy, while 6 died after sudden cardiopulmonary arrest. 

Results of HIF-1α and troponin levels according to PE clinical risk scoring are shown in Table 1.

**Table 1:** Comparison of the groups HIF-1 α, troponin-ı, ECHO and length of hospital stay

	Control group (mean ± SD)	Intermediate low risk PE (mean ± SD)	Intermediate high risk PE (mean ± SD)	High risk PE (mean±SD)	P*
HIF-1 α baseline (ng/mL)	0.46 ± 0.15*	0.65 ± 0.17*	0.95 ± 0.31*	1.35 ± 0.53*	0.001
HIF-1 α 72 hours (ng/mL)		0.47 ± 0.13	0.48 ± 0.27	0.41 ± 0.16	>0.05
Troponin-ı baseline (pg/mL)	10.2 ± 3.6*	16.7 ± 3.64*	801.9 ± 481.9*	1286.2 ± 714.2*	0.001
Troponin-ı 72 hours (pg/mL)		7.8 ± 5.6*	167.1 ± 155.1*	283.4 ± 249.9*	0.001
Systolic PAP (mm_Hg)		43.5 ± 6.91*	52.5 ± 8.38*	63.75 ± 16.37*	0.001
Length of stay (day)		4.72 ± 1.63*	8.95 ± 2.54*	11.31 ± 2.77*	0.001

P*: In the ANOVA between the groups, the average of high-risk PE group was significantly higher than the other groups.

Comparisons of HIF-1α levels between the patient groups showed that the high-risk PE group had significantly higher HIF-1α levels compared to the intermediate PE groups (P = 0.001 for both). When compared with the control group, the patient groups had significantly higher HIF-1α levels (P = 0.001 for all), while there were no significant differences in terms of 72-h HIF-1α levels (P = 0.273 for high-risk PE, P = 0.98 for both intermediate PE groups). Comparison of HIF-1α and troponin levels in the intermediate high-risk PE patients given thrombolytic therapy and those treated with enoxaparin sodium showed that HIF-1α levels were significantly higher in the group that received thrombolytic therapy (P = 0.001), while there was no difference in troponin levels (P = 0.146) (Table 2).

**Table 2:** Comparison of baseline HIF-1α and troponin levels intermediate high-risk PE patient treatment groups and between surviving and nonsurviving high-risk PE patients.

	Intermediate high risk PE DMAH treatment (n:42) (mean ± SD)	Intermediate high risk PE Thrombolytic treatment (n:18) (mean ± SD)	High risk PE Survival (n:39) (mean ± SD)	High risk PENon-survival (n:21) (mean ± SD)	P*
HIF-1 α baseline (ng/mL)	0.78 ± 0.11	1.35 ± 0.28	0.98 ± 0.21	1.52 ± 0.36	0.001/0.001
Troponin-ı baseline (pg/mL)	776.2 ± 544.2	862.3 ± 293.5	1174.3 ± 649.2	1198.2 ± 802.2	0.146/0.72

P*: Comparison between treatment groups/comparison of surviving and nonsurviving patients

The 21 patients who died due to high-risk PE had significantly higher mean HIF-1α level compared to survivors in the same patient group (P = 0.001). Troponin levels in the deceased and surviving patients in the high-risk PE group were not statistically significant (P = 0.72) (Table 2). This statistical analysis was not performed in the intermediate PE group due to the low mortality rate among those patients.

**Figure 1:**
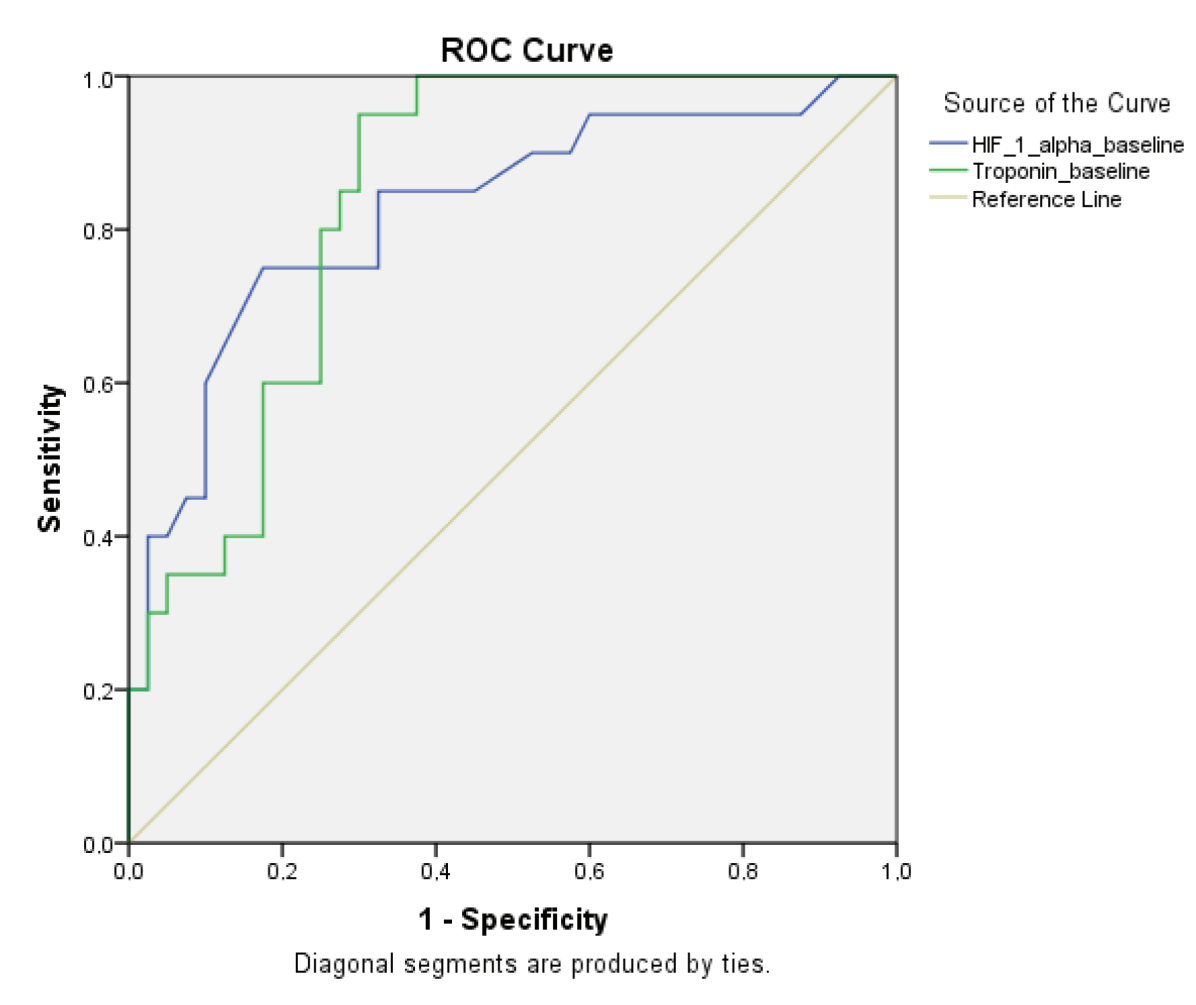
ROC curve analysis in patients with and without high-risk embolism with HIF-1 α, troponin-ı baseline HIF-1 alpha: Hypoxia inducible factor-1α

In the ROC curve analysis based on baseline HIF-1α and troponin levels of patients with and without high-risk PE, the areas under the curve were 83% and 84%, respectively. At a baseline level of 0.81 ng/mL, HIF-1α had a sensitivity of 85% and specificity of 78% in detecting high-risk embolism. Troponin showed sensitivity and specificity of 80% and 75%, respectively, at a baseline level of 715.9 pg/mL (Figure 1).

The high-risk PE patients had a mean pulmonary arterial pressure (systolic PAP) of 63.75 ± 16.37 mmHg and their mean length of hospital stay was 11.31 ± 2.77 days. These values were 52.5 ± 8.38 mmHg and 8.95 ± 2.54 days in the intermediate high-risk PE group and 43.5 ± 6.91 mmHg and 4.72 ± 1.63 days in the intermediate low-risk PE group, respectively. Systolic PAP and length of hospital stay were significantly greater in the high risk PE group compared to the intermediate high-risk PE group (P = 0.03 and P = 0.05, respectively) and in the intermediate high-risk PE group compared to the intermediate low-risk PE group (P = 0.001 for both) (Table 1). 

**Figure 2:**
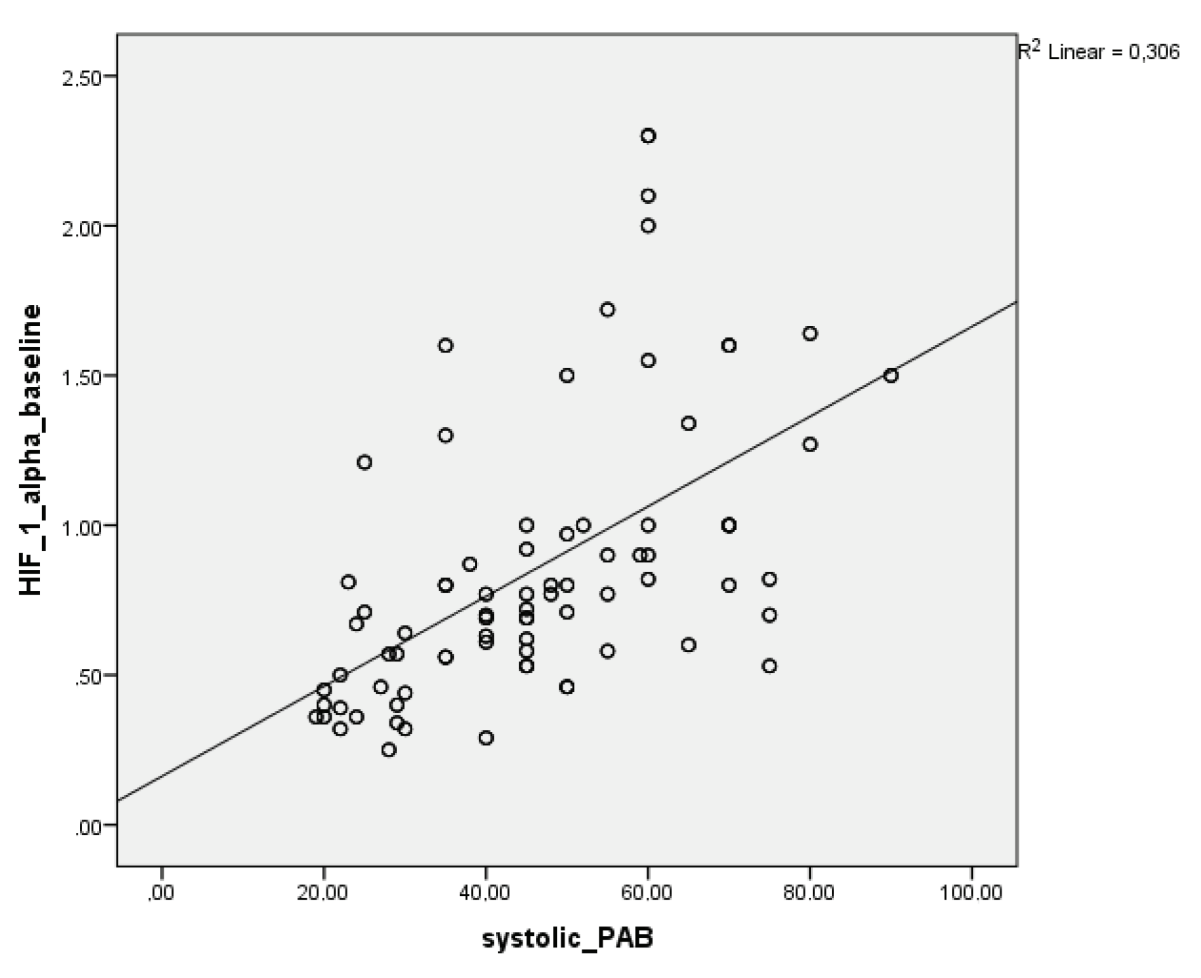
Correlation between HIF-1 alpha baseline and systolic PAB PAB: Pulmonary arterial pressure

**Figure 3:**
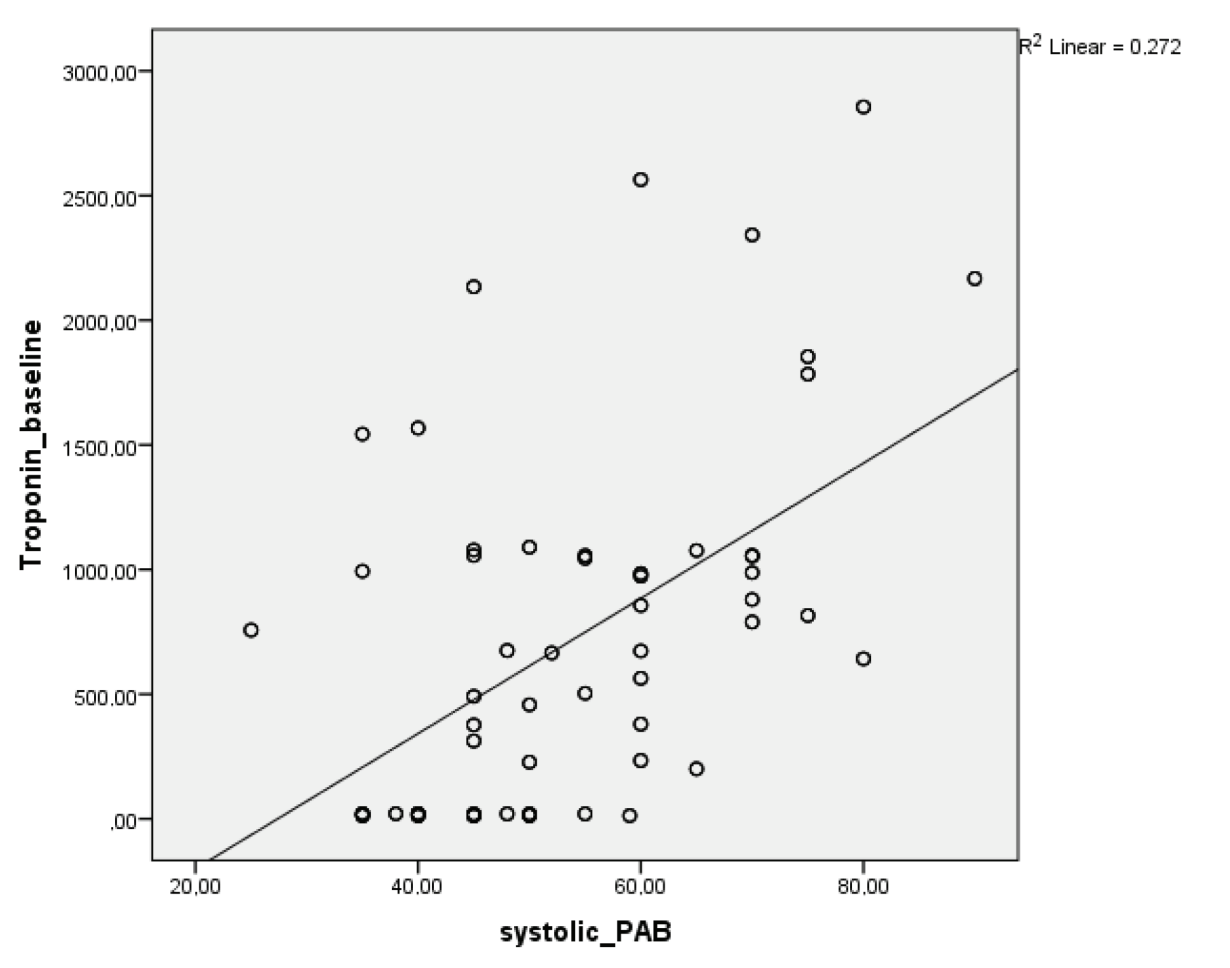
Correlation between troponin-ı baseline and systolic PAB PAB: Pulmonary arterial pressure

Baseline HIF-1α and troponin-I levels were moderately correlated with systolic PAP in patients presenting with PE (r = 0.553, P = 0.01; r = 0.521, P = 0.01). At 72-h follow-up, these correlations were weaker but still significant (r = 0.21, P = 0.01; r = 0.269, P = 0.01) (Figures 2 and 3).

## 4. Discussion

In this study, we observed that the HIF-1α levels of PE patients increased with higher PE clinical risk stratification in correlation with troponin-I level, one of the variables included in clinical scoring. Patients that required thrombolytic therapy or died had higher HIF-1α levels than other patients, whereas troponin-I values did not differ significantly in these groups. Therefore, HIF-1α may be a better prognostic marker than troponin-I.

The clinical presentations of PE are classified as high-, intermediate-, and low-risk [8]. In acute intermediate-risk PE patients, transthoracic ECHO is the most frequently used method to evaluate right ventricular dysfunction. Right ventricular dysfunction is defined using parameters such as right ventricular dilation, hypokinesis of the right ventricular wall, paradoxical movement of the interventricular septum, tricuspid valve insufficiency, increased pulmonary artery diameter, and higher ratio of right ventricular diastolic end diameter to left ventricular diastolic diameter [9,10]. 

Cardiac troponin T and I are enzymes specific to the cardiac muscles. Right ventricular dilation that occurs as a result of acute right heart failure due to high-risk PE increases right ventricle oxygen demand. Troponin, BNP, and NT-proBNP measurements may be useful in distinguishing high- and intermediate-risk patients from low-risk patients for prognostic evaluation. They can also be used to further stratify intermediate-risk patients into high-intermediate and low-intermediate-risk subgroups. However, it must be kept in mind that these markers are also elevated in conditions such as acute exacerbation of COPD, sepsis, acute kidney failure, trauma, rhabdomyolysis, and congestive heart failure [11,12]. Although cardiac biomarkers have an important place in clinical risk scoring, the absence of a definite cut-off value for the differentiation of high-risk and high-intermediate-risk patients necessitates clinical observation for thrombolytic therapy in the high-intermediate-risk group. However, the time spent in clinical follow-up sometimes works to the patient’s detriment, with hypoxic respiratory failure and sudden hemodynamic collapse resulting in a precipitous death. This led to the need to investigate alternative biomarkers in clinical scoring.

The *HIF1* gene, which is activated under hypoxic conditions, functions by hydroxylating and activating the proline hydroxylase α subunit. Primarily through erythropoietin synthesis, HIF-1α prevents reoxygenation injury via VEGF-mediated neovascularization, glycolytic activity, and glutathione peroxidase. It activates enzymes that prepare cells to withstand hypoxia. 

In rat models of induced PE, HIF-1α mRNA expression was detected in the pulmonary arterial wall. In contrast, HIF-1α expression was not observed in samples of human myocardial tissue from healthy individuals at the onset of right ventricular hypertrophy but increased in correlation with myocardial ischemia. In a rat model of induced PE, HIF-1α level was found to increase with the onset of disease, show rapid elevation until 72 h in the untreated period, then increase at a slower rate until week 4 [13]. In another study of recombinant adenovirus-mediated HIF-1α in a rat model, it was observed that HIF-1α increased VEGF-A level, which plays an important role in thrombus organization and thrombolysis [14]. It was observed that HIF-1α, the main inducer of VEGF synthesis, also plays an important role in transmural angiogenesis in heart failure in addition to the effects of VEGF on thrombi [15].

Based on the results of our study, we observed significant differences in baseline levels of HIF-1α and troponin-I between all groups. However, in the intermediate-risk group, who were the main focus of this study, our observation of a significant difference in baseline HIF-1α but not in troponin-I between the 18 patients who received thrombolytic therapy and the 42 patients who were followed with low-molecular-weight heparin suggests that HIF-1α may be an important criterion for implementing early reperfusion therapy in this patient group. ROC curve analysis between HIF-1α and troponin-I levels showed that although the sensitivity and specificity of HIF-1α were relatively low, they were still higher than those of troponin-I in the distinction of high-risk PE. Using the two tests together in the discrimination of massive PE (with cut-off values of 0.81 ng/mL for HIF-1α, and 715.9 pg/mL for troponin-I) resulted in 68% sensitivity and 94% specificity. On the other hand, if a test was considered positive, the sensitivity was 98% and the specificity was 69%. Furthermore, the higher HIF-1α levels among deceased patients compared to survivors of PE might demonstrate the utility of this parameter as an early indicator of poor prognosis.

Mortality in the present study was limited to 21 patients in the high-risk PE group and 9 patients in the intermediate-high-risk PE, which may increase the margin of error in the early recognition of poor prognosis. For this reason, more extensive studies with larger patient groups are needed.

In conclusion, HIF-1α may provide an early guide for clinical risk stratification of PE and the administration of thrombolytic therapy to early intermediate-high-risk patients. Initial HIF-1α is a better predictor of mortality than troponin level and thus may be a more useful parameter in clinical follow-up.

## Conflict of interest

The authors declare that they have no conflict of interest.
